# The Cesarean Operation under Difficulties

**Published:** 1907-07

**Authors:** Frank Thomas Woodbury

**Affiliations:** Gandara, Island of Samar, P. I. Captain and Assistant Surgeon, U. S. Army


### SELECTED ARTICLES.

### The Cesarean Operation Under Difficulties.

By FRANK THOMAS WOODBURY, Gandara, Island of Samar, P. I.
Captain and Assistant Surgeon, U. S. Army.

[*New York Medical Journal, May 4, 1907.*]

IN the late afternoon of December 20, 1906, a native Visayan urgently requested me to come and see his wife, who was in labor, as he feared matters were not going well. I thought that possibly a brief report of this case may not be uninteresting to the readers of the *Journal.*

Gandara is a one company post, which is fifteen miles from the west coast of the island of Samar, and is situated on a peninsula, at the junction of the east and north forks of the Gandara River, a noble stream flowing through a wild, uncultivated land of mountain and jungle. This waterway, owing to the absence of woods and even trails through the dense vegetation, is the only highway from the interior of Samar to the coast. Going on board the launch, which in our isolated position affords our only means of communication with civilisation and the outside world, I went down to Dumaloöng, which is a native barrio, or village, even more miserable in its squalor and poverty than many similar native towns in other islands. In passing I may remark that inhabitants of Samar are perhaps the most worthless, lazy, and unenterprising people in the whole archipelago. This town is merely a succession of filthy, ramshackle huts of bamboo and *nipa* (palm leaf) thatch built along the river bank in a double row. The space between is dignified by the name of street; but it is nothing save the soil worn bare of grass by the feet of pedestrians. As it rains almost all the time in Samar, the avenue is naught but a collection of bogs and sloughs, which the people hop over, or walk around, and in which the ubiquitous native razorback pig grunts and wallows. The inhabitants live upon rice, a poor variety of small fish which is usually regarded as unfit for food, a few eggs, a chicken now and then, and once in a while, as a great treat, they kill one of their half-starved pigs.

Their clothing consists of two or three garments, and also a pair of slippers, if they are well to do.

Having landed at this metropolis in the dusk of the evening, by a bamboo staging so rickety and broken that I nearly fell through, I proceeded to the house. This resembled a large barn in shape and appointments, being used as a store house for *abacá* (the famous manila hemp fibre) and *palay,* or unhusked rice.

Having fought our way through the usual crowd of cur dogs, and passed through the doorway, we encountered about fifty natives squatting in the house, smoking cigarettes and talking like a flock of parrots.

One corner of the floorspace, about eight feet square, had been screened off with matting; thus making a square room, lined inside with sheets and covered with mosquito bar. This small apartment was imperfectly lighted by an oil lamp and two cocoanut oildips. Here I found the patient dressed in her usual clothing, consisting of a chemise and a print skirt, lying upon mats on the floor and with two native cotton pillows under her head. One woman was holding her hands, another her feet, and a man was pressing down with his fists in the pit of her stomach; while around her waist, a rope of twisted sheet had been tightly wound. This is the usual course of procedure among the Visayans of Samar when any one is sick.

Two other native men came in with the husband and myself, and all squatted sociably upon the floor around the patient. The husband, who fortunately understood Spanish, interpreted for me. I first had the windows (which were nothing but palm leaf shutters) opened, as the air was almost stifling, it was so hot. I then ordered every one out but the husband and two women, and removed the noose from the waist.

After much difficulty I learned that the woman was thirty-three years of age, and that this was her first confinement. (This is rather an advanced age for a native primipara.) The period of gestation could not be fixed, owing to ignorance of the last menstrual date; guesses were made averaging from six to twelve months. They then informed me that she had been in labor only since eight o'clock in the morning, but I learned several days later that she had been in labor thirty-six hours when I arrived.

Examination showed the anemic, fragile frame usual to native women. Lungs and heart normal, pulse a little rapid, respirations normal, breasts firm, nipples prominent with dark areolæ. The uterus extended to two finger breadths above umbilicus; fetal heart sounds were faintly audible, child in left occipitoanterior position. Vaginal examination by speculum showed a widely dilated os. The liquor amnii had escaped and the head was firmly wedged between pubis and promontory of the sacrum. The anteroposterior diameter of the pelvis seemed smaller than normal.

As I was told that she had been in labor only about ten hours then and seemed to be in fair condition, I decided to wait and observe the pains and determine more surely if the narrowing of the superior strait was sufficient to prevent birth. My observations were cut short by the sudden onset of a convulsion. Here was a dilemma. The uterus had not once contracted forcibly during my presence, and the surroundings of the patient combined with the lack of skilled assistants, clearly showed the impossibility of obtaining asepsis; while the contracted pelvis forbade attempts at podalic version.

There was no forceps nearer than eight hours by launch, and even had this instrument been available the pelvic narrowing would have no doubt prevented its successful application. Attempts at manual traction on the head with external kneading of the uterus failed utterly. Chloroform was promptly administered at the onset of the convulsive attack, which soon produced its abatement.

A quick decision was made as a last resort to perform a Cesarean section. By this time an American teacher in the agricultural school arrived, and explained the crisis in fluent Visayan to the husband and relatives, who consented to seize the offered hope of success. Accordingly, after the priest had been summoned, the woman still partly under chloroform was wrapped in blankets, placed on a cot, and carried down to the launch by six natives. The procession created quite an excitement among the pigs, dogs, and populace.

The boat took twenty minutes to arrive at the post from whence I had been called, and the patient was immediately transferred to the small military hospital, the procession of patient, doctor, priest, and anxious relatives making a memorable picture in the tropical moonlight.

The hospital building is a long, low structure, of materials and workmanship similar to all native houses, but with an Oregon pine board floor. The rafters are bare, and the individual rooms are merely divided off from the large room by rough boards. The room used for operating was 10 feet long by 12 feet wide, and lit by an oil lamp. My only available assistants were a sergeant first-class, and two privates of the Hospital Corps. I immediately put my instruments and suture materials to boil on the kitchen stove; and also sterilised a large boiler full of water. The patient was given a hypodermic injection of atropine 1|200, strychnine 1|30 grain, which was subsequently repeated during the operation. Private Spofford, of the Hospital Corps, administered the chloroform under my direction.

The field of operation was thoroughly scrubbed with tincture of green soap, hot water, and a hand scrub brush for five minutes, then washed with alcohol and a gauze sponge, followed by hot sterile water, and finally rinsed with alcohol again. The intended line of incision was thoroughly painted with tincture of iodine from three inches above the umbilicus down to the pubis in the median line. Sterile towels were placed over the abdomen, and my chief assistant, Sergeant Doran, and I sterilised our hands by scrubbing for five minutes with tincture of green soap, hot water, followed by alcohol, potassium permanganate, oxalic acid, and finally with sterile water. The instruments now being ready, the suture material was transferred from the sterilizer to a tincture of iodine.

A median incision was made over the iodine line from two inches above the navel, to two inches above the pubis. The fundus of the uterus was delivered through the wound, the intestines being kept back by gause pads wet with hot, normal salt solution. The uterus was then rapidly opened in a line corresponding to the muscle incision, with scissors. The placenta was found on the anterior wall. A living male child was extracted. The sergeant then firmly grasped the neck of the uterus controlling the uterine vessels. The cord was clamped, and the baby turned over to Private Betononay, Hospital Corps, who wrapped it up in a warm blanket as soon as respiration was established. The uterus was immediately emptied of placental membranes and clots, and the wound closed with continuous sutures of iodised raw silk, passing down to, but not through, the uterine mucosa. The peritoneum was closed similarly, and the abdominal parietes with interrupted iodised silkworm gut sutures by means of a Riverdin needle. A gause dressing and abdominal binder were applied; a partial enema of normal salt solution was given, and the patient put to bed with hot blankets and hot water bottles. The entire time consumed in preparation and operation was forty-five minutes.

The patient rallied well and speedily from the anesthetic. One hour after operation the temperature was 100° F., pulse 136, respiration 36. Hot water in teaspoonful doses was allowed to quench thirst. The pulse, temperatures, and respirations reached their highest the day following, being 102° F., 137, and 34, at 3 p. m. After this, they fluctuated, coming down to normal on the eighth day and remaining so until the patient was discharged from the hospital on January 8th, the nineteenth day after operation. Atropine 1|200, strychnine 1|30 grain were given, at first every four hours, and morphine at night when needed, hypodermically. Later they were given by mouth with acetphenetidin 3 grains.

Urine was passed by catheter every four hours until the third day, and showed no albumin at any time (specific gravity 1.020 to 1.027). The bowels were kept open by enemata of magnesium sulphate ounce 1, glycerin drachms 4, oil of turpentine drops 5, in a pint of hot water, and, later, by compound aloin pills, administered on two occasions. The uterus and vagina were douched daily with Lugol's solution ounce 2, in hot normal salt solution two quarts, and the vagina packed with iodoform gause. The day following the operation, the patient vomited a quantity of bilious fluid mixed with rice. On the second day she was allowed her first nourishment in the shape of 8 c.c. of sherry wine with 30 c.c. of beef tea, given at three hour intervals with a little sodium bicarbonate. The wound healed by first intention, and the stitches were removed in two sittings, on the twelfth and fourteenth days.

An abdominal binder made to fit and button on was applied and directed to be worn for the next eighteen months. The patient made a steady improvement from the third day. The breasts, owing to the atropine and gentle massage, gave very little discomfort; the secretion being very scanty on the day of discharge. The baby was turned over to a native woman, who volunteered to act as wet nurse. With usual native indifference, she failed to obey orders, and I did not see the infant again. Its death was reported later.

Considering the lack of facilities, the unfavorable surroundings, the gravity of the operation, the unskilled assistants, and the kind of nursing, which although most willing and painstaking, was inexperienced in this class of cases, and especially the woman's natural state of nitrogen starvation and secondary anemia (which is seen in most women in the archipelago, owing to their exclusive diet of rice and fish), the happy termination of this case seems nothing less than a triumph of modern surgery.

